# The Early Adhesion Effects of Human Gingival Fibroblasts on Bovine Serum Albumin Loaded Hydrogenated Titanium Nanotube Surface

**DOI:** 10.3390/molecules26175229

**Published:** 2021-08-28

**Authors:** Yuchen Sun, Ran Lu, Jingming Liu, Xin Wang, Haitao Dong, Su Chen

**Affiliations:** Laboratory of Biomaterials and Biomechanics, Beijing Key Laboratory of Tooth Regeneration and Function Reconstruction, School of Stomatology, Capital Medical University, Beijing 100050, China; sunyuchen0718@sina.com (Y.S.); 13521320288@163.com (R.L.); mingyan1112@163.com (J.L.); angus.wangxin@outlook.com (X.W.); jay41dong@126.com (H.D.)

**Keywords:** nanotubes, drug-loaded, HGFs, adhesion

## Abstract

The soft tissue sealing at the transmucal portion of implants is vital for the long-term stability of implants. Hydrogenated titanium nanotubes (H_2_-TNTs) as implant surface treatments were proved to promote the adhesion of human gingival fibroblasts (HGFs) and have broad usage as drug delivery systems. Bovine serum albumin (BSA) as the most abundant albumin in body fluid was crucial for cell adhesion and was demonstrated as a normal loading protein. As the first protein arriving on the surface of the implant, albumin plays an important role in initial adhesion of soft tissue cells, it is also a common carrier, transferring and loading different endogenous and exogenous substances, ions, drugs, and other small molecules. The aim of the present work was to investigate whether BSA-loaded H_2_-TNTs could promote the early adhesion of HGFs; H_2_-TNTs were obtained by hydrogenated anodized titanium dioxide nanotubes (TNTs) in thermal treatment, and BSA was loaded in the nanotubes by vacuum drying; our results showed that the superhydrophilicity of H_2_-TNTs is conducive to the loading of BSA. In both hydrogenated titanium nanotubes and non-hydrogenated titanium nanotubes, a high rate of release was observed over the first hour, followed by a period of slow and sustained release; however, BSA-loading inhibits the early adhesion of human gingival fibroblasts, and H_2_-TNTs has the best promoting effect on cell adhesion. With the release of BSA after 4 h, the inhibitory effect of BSA on cell adhesion was weakened.

## 1. Introduction

Peri-implantitis is the most common complication of oral implantation, with incidence of 8.9–43.3% [1]. Previous studies proposed that the long-term stability of oral implant could be promoted by enhancing osseointegration. However, a large number of studies found that soft tissue sealing at the transmucosal part of implants played a particularly important role in reducing the inflammation around the implant, which could enhance the stability of the implant as well. The soft tissue around the implant is weaker than that of natural teeth, which is more prone to the penetration of bacteria [2]. If the soft tissue sealing is enhanced, the risk of bacteria invasion and apical migration of the junctional epithelium can be reduced, which can further lower the risk of peri-implant inflammation [3,4]. According to Gristina’s theory of surface competition [5], soft tissue cells and oral bacteria compete for the limited binding sites on the biomaterials surface; therefore, increased numbers and diversity of soft tissue cells is considered to reduce the adhesion of bacteria at an early time point. Therefore, many studies have focused on surface modification of biomaterial to enhance the bioactivities of soft tissue cells [6].

Titanium dioxide nanotubes (TNTs) are a surface modification method producing uniform and stable nanotubes on the surface of titanium. TNTs were proved to have strong photocatalysis [7,8], nanoscale morphology with high surface energy, and were widely used as drug carriers with therapeutic potential due to the tube structure [9]. Proteins [10], metal ions [11], and growth factors [12] have been tested and loaded in nanotubes. Particle concentration on the surface of the biomaterial could be maintained at a specific value for a long time, which could facilitate bioactivities of cells or provide anti-inflammatory effects. However, not all loaded particles could provide positive effects to tissue. Cytotoxicity, genotoxicity, and inhibition of high concentration of proteins to local tissue should also be considered. Our previous studies that examined hydrogenated titanium nanotubes (H_2_-TNTs) by thermal treatment with hydrogen on TNTs [13] demonstrated that H_2_-TNTs also had the capabilities of protein-loading and protein-releasing, and the capability of promoting initial adhesion of human gingival fibroblasts (HGFs) due to their super hydrophilic surface [14]. However, there had been no further research about whether the protein-loaded H_2_-TNTs could promote the adhesion of HGFs around implants.

Bovine serum albumin (BSA) is often used as a loading protein because its structure is similar to that of human serum albumin [15], and it is the most abundant albumin in body fluid [16]. As the first protein arriving on the surface of the implant, albumin plays an important role in the initial adhesion of soft tissue cells [17]. A previous study [10] also exhibited that BSA could promote the early adhesion of HGFs when delivered by a sustained release system. Due to the cycling, nutritional, and physiological functions of BSA, it is also a common carrier, transferring and loading different endogenous and exogenous substances, ions, drugs, and other small molecules [18]. BSA is used as the loading protein in the present study and can pave the way for the future experiments investigating the loading capability of other particles.

The purpose of this study was to investigate whether BSA-loaded H_2_-TNTs could promote the early adhesion of HGFs and promote soft tissue closure at implants. The hypothesis of this study was that BSA-loaded H_2_-TNTs have beneficial surface properties, protein-releasing function, and could accelerate the early adhesion of HGFs.

## 2. Results

### 2.1. Sample Characterization

**Figure 1:**
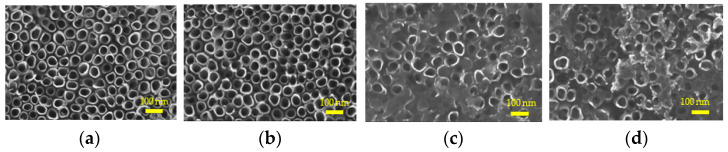
SEM images of (**a**) NT (TNTs); (**b**) HNT (H_2_-TNTs); (**c**) BNT (BSA-loaded TNTs); (**d**) BHNT (BSA-loaded H_2_-TNTs).

The morphology of each sample was observed by SEM (Figure 1). A uniform and stable nanotube array was organized on the surface of NT and HNT, with the diameter of 100 nm. After BSA was loaded into the nanotubes, BSA was further adsorbed on the surface of the material in the form of films.

The results of XPS (X-ray photoelectron spectroscopy) showed that N1s and S2p could be seen on the surface of BNT and BHNT, which were the characteristic elements of albumin (Table 1).

**Figure 2:**
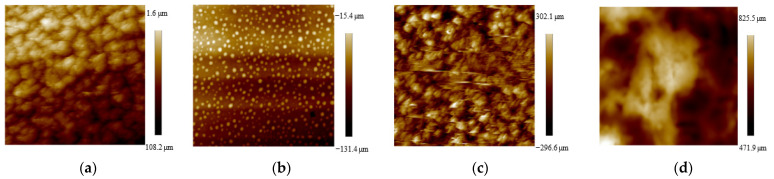
AFM results of (**a**) NT; (**b**) HNT; (**c**) BNT; (**d**) BHNT.

**Figure 3:**
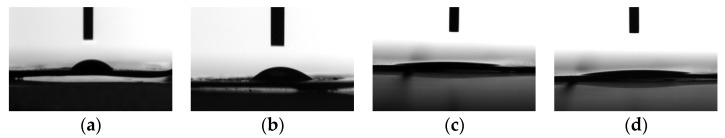
Contact angles of (**a**) NT; (**b**) HNT; (**c**) BNT; (**d**) BHNT.

The roughness (Ra) and contact angle (CA) of the substrates were measured (Figure 2 and Figure 3, Table 2). The Ra of NT and HNT were 98.0 and 98.7 nm, respectively. After BSA was loaded, the Ra of BNT and BHNT increased to 122.1 and 148 nm, respectively. The CA of NT was 46.2°, while the CA of BNT decreased to 36.4°. The surface of HNT formed a super hydrophilic surface with a CA of 7.5°. After BSA was loaded, the CA of BHNT increased to 9.4°, though this was not a significant difference.

### 2.2. The Capability of Protein-Loading and Protein-Releasing

**Figure 4:**
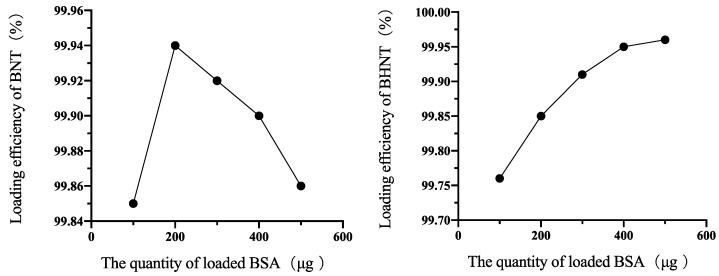
BSA loading efficiency of BNT and BHNT.

The 100, 200, 300, 400, and 500 μg BSA was loaded into nanotubes of NT and HNT (Figure 4). The efficiency of BHNT increased with the increasing quantity of loading BSA. While the highest loading efficiency of BNT was 99.94% when loading 200 μg BSA, the loading efficiency gradually decreased with the increase of BSA loading.

**Figure 5:**
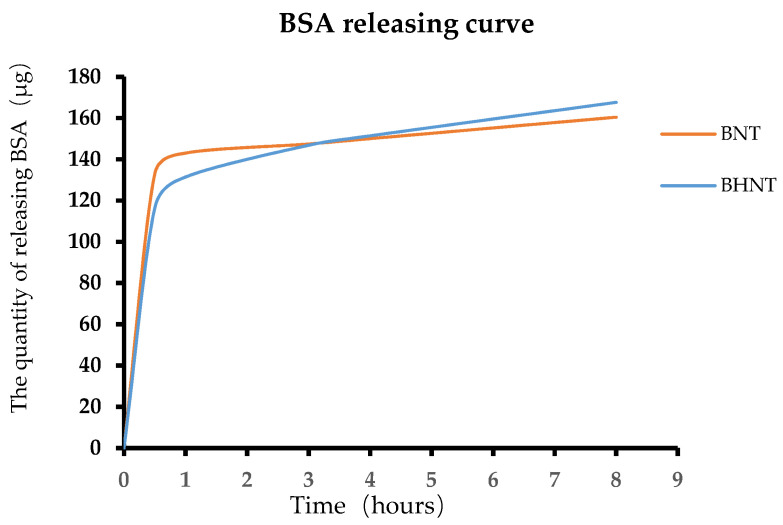
BSA releasing curve of BNT and BHNT.

Shown in Figure 5, both BNT and BHNT exhibited a high-rate release of BSA over 1 h then followed by a period of slow and sustained release. When loading the same quantity of BSA, the quantity of released BSA of BNT and BHNT was 143 and 131.4 μg at 1 h and the quantity of released BSA of BHNT was slightly higher than BNT at 4 h.

### 2.3. Response of HGFs to the Substrates

#### 2.3.1. Morphology of HGFs

**Figure 6:**
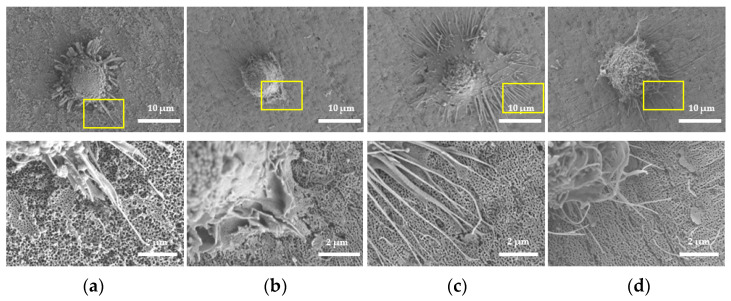
SEM analysis of cell morphology at 1 h of (**a**) NT; (**b**) HNT; (**c**) BNT; (**d**) BHNT.

**Figure 7:**
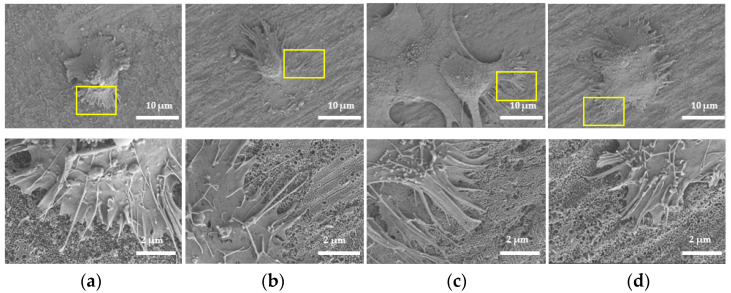
SEM analysis of cell morphology at 4 h of (**a**) NT; (**b**) HNT; (**c**) BNT; (**d**) BHNT.

After being cultured for 1 h (Figure 6), the morphologies of HGFs on NT, BNT, and BHNT were spherical, with only few filopodia protruding, while HGFs on HNT were flat, with a larger extension range and more filopodia protruding than in the other three groups. High-magnification micrographs of HGFs on HNT showed that filopodia extended into the nanotubes, forming a tighter combination. HGFs grew flat and were accompanied with lamelliopodia after being cultured for 4 h (Figure 7). Compared with the other two groups, HGFs on HNT and BHNT had a wider range of cell adhesion and extension, and more lamelliopodia protruding. HGFs on HNT formed focal adhesion at 4 h, which indicates better adhesion than in other groups. High-magnification microscope images showed that more pseudopodia were anchored and extended into the nanotubes on HNT and BHNT.

#### 2.3.2. Assay of Adhered HGs

**Figure 8:**
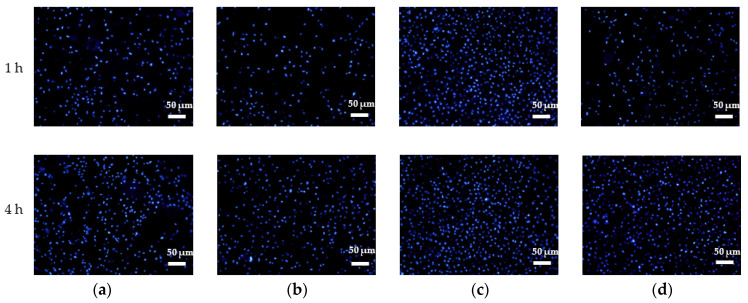
Nuclei immunofluorescence staining by DAPI. HGFs cultured on (**a**) NT; (**b**) HNT; (**c**) BNT; (**d**) BHNT for 1 and 4 h.

**Figure 9:**
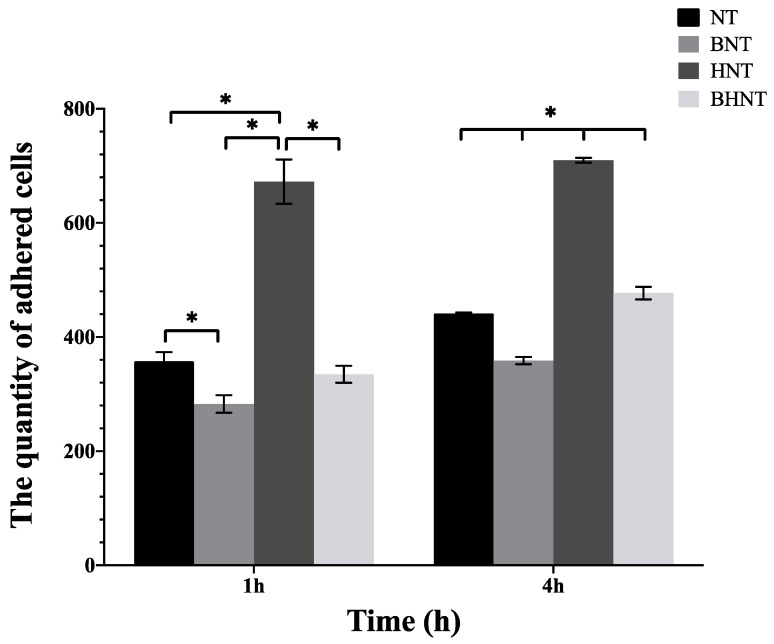
Quantity of adhered HGFs cultured for 1 and 4 h. * *p* < 0.05.

Through immunofluorescence staining and ImageJ software analysis (Figure 8 and Figure 9), we determined that there was a large quantity of HGFs adhered on HNT at 1 h, and the number of BNT and BHNT was less than that of NT and HNT, the loading of BSA inhibited the early adhesion of HGFs on both BNT and BHNT. After 4 h, the number of adhered fibroblasts on HNT was also significantly higher than that of NT and the number of adhered fibroblasts on BHNT was significantly higher than that of NT or BNT.

#### 2.3.3. RT-qPCR

**Figure 10:**
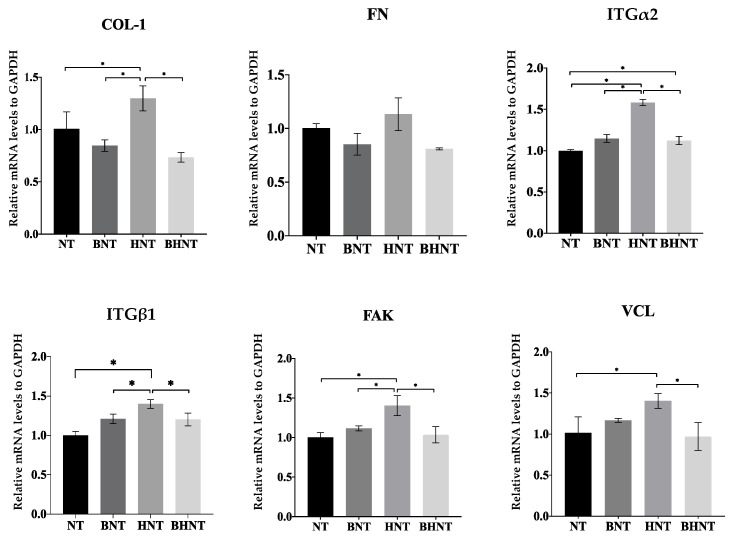
RT-qPCR detection of adhesion-related gene expression of HGFs cultured on the samples for 1 h with statistical significance determined by *p* < 0.05. Expression of COL-1, FN, ITGα2, ITGβ1, FAK, and VCL was determined by the relative amount of mRNA with formula 2 ^(−ΔΔCt)^. * *p* < 0.05.

**Figure 11:**
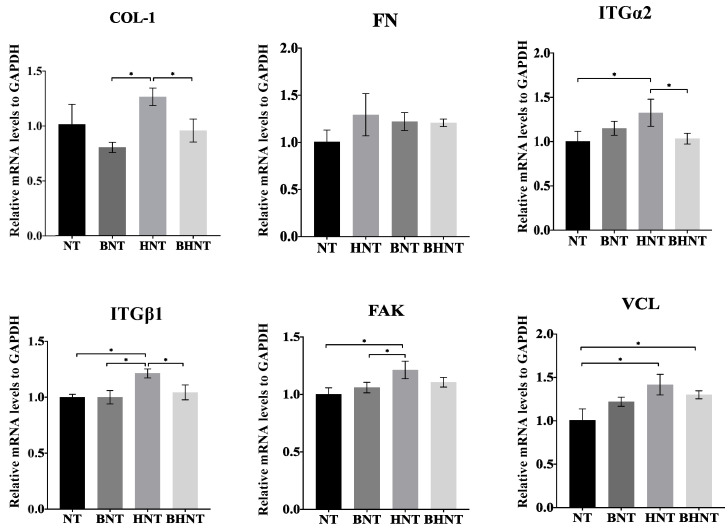
RT-qPCR detection of adhesion-related gene expression of HGFs cultured on the samples for 4 h with statistical significance determined by and *p* < 0.05. Expression of COL-1, FN, ITGα2, ITGβ1, FAK, VCL was determined by the relative amount of mRNA with formula 2^(−ΔΔCt)^. * *p* < 0.05.

mRNA expression levels of adhesion related proteins were measured (Figure 10 and Figure 11). When cultured for 1 h, HGFs on HNT showed higher mRNA expression levels of COL-1, ITGα2, ITGβ1, FAK, and VCL than the other groups (*p* < 0.05). At 4 h, BHNT showed higher expression of ITGα2, ITGβ1, FAK, and VCL than BNT, but no significant differences were observed (*p* > 0.05).

## 3. Discussion

In the present study, BSA was loaded in H_2_-TNTs by vacuum drying to combine surface modification and drug-releasing techniques to promote the bioactivity of HGFs. Hence, a comprehensive evaluation was used to investigate the effects of BSA-loaded H_2_-TNTs in this study. However, BSA-loaded (hydrogenated or non hydrogenated) TNTs inhibited the early adhesion of HGFs cells.

BSA adsorbed on the surface formed the sudden burst release at the early stage, which temporarily increased the protein concentration in the microenvironment of the contact part between the biomaterial surface and cells, thus affecting the adhesion of HGFs. Previous studies exhibited that cells secreted small molecular proteins which covered the surface of the material before they adhered to the biomaterial surface, then the cells adhered to the protein layer, and according to the Vorman effect, only after the periodic fracture of the protein adsorbed on the surface of the material will new protein molecules occupy the binding site [19,20]. When the secretory protein contacted the surface of the material with the state of high concentration BSA, the secretory proteins of BNT and BHNT needed to replace a large number of small molecular proteins on the surface of materials. Therefore, the number of adhered cells on BNT and BHNT were less than the group without BSA at 1 and 4 h. Whereas, some researchers showed that TNTs loaded with BSA promoted adhesion of HGFs better than TNTs at 1 and 3 h [10], which is likely due to the differences of the total quantity of loaded BSA and releasing rate with us. Our previous studies also demonstrated that the super hydrophilicity of H_2_-TNTs could promote the adhesion of HGFs, but the present study showed that BSA-loaded H_2_-TNTs had greater inhabitation than H_2_-TNTs because the sudden releasing effects may ameliorate the effect brought by morphology, though the surface is H_2_-TNTs.

Moreover, H_2_-TNTs had higher loading efficiency than TNTs in the present study. The drug solvent mainly penetrated the nanotubes by capillary force [21], and the permeability of the nanotubes could be enhanced by increasing the hydrophilicity of material surface. H_2_-TNTs could load a higher quantity of BSA than TNTs because the super hydrophilic nanotubes could load BSA more deeply. The primary effect of albumin adsorbed into the nanotubes was the increase in entropy, while high surface energy and negative surface charge could also affect the releasing effect [22,23,24,25,26]. The super hydrophilic surface of the H_2_-TNTs had high surface energy and the large amount of -OH on the surface provided greater affinity for BSA, which could bind BSA more closely. Therefore, BSA-loaded H_2_-TNTs had slower release effecicency than that of BSA-loaded TNTs and the BSA releasing curve of BSA-loaded H_2_-TNTs was gentler before 4 h.

The surface properties of biomaterials [27] have a crucial effect on cell adhesion, proliferation, and other biological behaviors. The formation of BSA film hindered the exposure of nanotubes and the effect of nanotube morphology on HGFs biological behaviors. BSA on the surface could not be completely removed because BSA, a negative charged protein, was adsorbed by electrostatic action. The hydrophilicity of TNTs was enhanced after BSA was loaded, while the super hydrophilicity of HNT was not further enhanced, because the adsorption of BSA affected the combination of -OH and water molecules. The results of SEM showed that the number and length of pseudopods on the surface of the two groups with BSA were lower after 1 h incubation, primarily because they were still in the protein recognition and replacement stage. Pierre et al. [28] explained that the extracellular membrane could sense surface morphology before cell adhesion. Due to the superhydrophilicity, HNT could bind water molecules and proteins at an earlier time point, and the pseudopodia could penetrate the nanotubes, forming a tight binding. The sudden release of BSA during the initial 1 h, causing a high concentration, inhibited the contact between the pseudopodia and the surface. However, after culturing for 4 h, HGFs on HNT and BHNT had more spread extension, which is a sign of migration and activation [29,30]. This cell adhesion behavior was mediated by the cytoplasm. A long and thin cytoplasm interacted with the surface, forming focal contact and stronger adhesion points [31,32,33], which played a key role in the reaction of HGFs adhesion.

The results of expression levels of mRNA showed the consistent trend as well except for FN. Fibronectin (FN) is one of the most important matrix adhesion proteins, which contributes to the initial adhesion of HGFs and the subsequent formation of the soft tissue seal [34]. As a macromolecular protein, FN expresses, secretes and replaces the small molecular protein only when small molecular proteins are completely adhered to the surface, which needed more time [14]. Therefore, there was no significantly high expression of FN in any group at 1 and 4 h. Integrin (ITG) is a member of the transmembrane glycoprotein family, which can optimize cell-to-cell and cell-to-substrate behaviors. Focal adhesion kinase (FAK) is a key signal kinase, linking integrin and the cytoskeleton in focal adhesion. The expression and phosphorylation of FAK are closely related to the mechanical conduction of cells in response to extracellular physical signals [35,36,37], which further mediates the expression of membrane protein-related genes in the process of cell adhesion, diffusion, and movement. Vinculin (VCL) is a component of adhesion plaques and adhesion junctions, and acts as an adhesion receptor for integrin. The key function of VCL is stimulation of actin polymerization and recruitment of protein remodeling proteins into the cytoplasmic tail of integrin [38,39], which regulates adhesion and achieves effective cell migration. As a signal pathway, ITG, FAK, and VCL showed the same expression trend at different time points.

Compared with other groups, the 1 h HNT group showed a higher expression trend, while there was no significant difference between BHNT and BNT, because of BSA. At 4 h, the expression of BHNT in COL, ITGβ1, FAK and VCL showed uptrend compared with the BNT group. The reasons may be as follows: first, with the release of BSA, a suitable concentration in the microenvironment was formed on the surface of the material to promote cell adhesion, second, with the release of BSA, the super hydrophilic surface of HNT was more conducive to further cell adhesion. Therefore, the expression of HGFs early adhesion protein related genes in BHNT showed an increasing trend, but no significant difference was found. The reason may be the close time interval. Therefore, further studies should allow for a longer time interval to determine whether HGFs adhesion and adhesion protein related genes expression were changed at more time points and explore the influence of other substances loaded on the surface of H_2_-TNTs on the early adhesion of HGFs.

Overall, the superhydrophilicity of H_2_-TNTs is conducive to the loading and sustained release of BSA. BSA-loaded (hydrogenated or non hydrogenated) TNTs have an inbitation effect on early adhesion of HGFs, but the effect of BSA-loaded H_2_-TNTs gradually decreased over time. It is necessary to increase the observation time and conduct more experiments to investigate the effect of BSA-loaded H_2_-TNTs on the adhesion and proliferation of HGFs.

## 4. Materials and Methods

### 4.1. Fabrication of the TNTs and H_2_-TNTs Arrays

Pure Ti (99.99%; Cuibolin Nonferrous Metal Industry Co., Ltd., Beijing, China, 10 × 10 × 0.2 mm^3^) was sequentially sonicated in acetone, ethyl alcohol, and deionized water for 10 min each. Anodization of Ti was conducted in the electrolyte, containing ethylene glycol (less than 0.5 wt% H_2_O), 0.5 wt% ammonium fluoride (NH_4_F), 10 vol% deionized water, at 20 °C 50 V for 15 min. The substrates were rinsed with ethanol, deionized water, dried, and then annealed at 500 °C for 2 h in air to obtain titanium nanotubes. TNTs were rinsed with deionized water, dried and sealed in a vacuum with hydrogen, and then annealed at 500 °C for 4 h to obtain the hydrogenated TNTs.

### 4.2. Loading BSA into the TNTs and H_2_-TNTs

The TNTs and H_2_-TNTs were rinsed with deionized water before BSA loading. Several qualities of BSA (100, 200, 300, 400, and 500 μg) were loaded into TNTs and H_2_-TNTs in order to determine the most appropriate BSA-loaded efficiency of each substrate. Then, 100 μg/mL BSA was dripped onto TNTs and H_2_-TNTs, covering the entire substrates without spilling. The BSA-loaded TNTs and H_2_-TNTs were dried under a vacuum at room temperature until the solution was no longer visible on the substrates. The above steps were repeated until specific qualities of BSA were loaded. After the BSA was loaded, the substrates were rinsed with 1 mL PBS (phosphate buffered saline) to remove unbounded BSA from the TNTs and H_2_-TNTs. The solutions were collected and analyzed with a BCA protein assay kit (Beyotime Institute of Biotechnology Co., Shanghai, China). The optical density (OD) at 562 nm was measured with a precision microplate spectrophotometer (SpectraMax Paradigm, Molecular Devices, CA, USA). The loading efficiency was analyzed according to the following equation:

(1) $$\eta =\frac{\left(Ql-Qs\right)}{Ql}$$

where *η* is the loading efficiency, *Ql* is the quality of the loading BSA, and *Qs* is the quality of BSA in solution.

TNTs with the highest efficiency of loading BSA were selected as BSA-loaded TNTs and H_2_-TNTs with the same efficiency were selected as BSA-loaded H_2_-TNTs, respectively. Triplicates of three independent samples were measured for each condition.

### 4.3. Characterizations of Samples

Four groups were examined: TNTs (NT), H_2_-TNTs (HNT), BSA-loaded TNTs (BNT), and BSA-loaded H_2_-TNTs (BHNT). The surface morphologies of NT, HNT, BNT, and BHNT were detected with a field-emission scanning electron microscope (SEM, S4800; Hitachi Ltd., Tokyo, Japan) for surface morphology observation. The chemical composition of the substrates was examined by X-ray photoelectron spectroscopy (XPS; ESCALAB 250Xi, Thermo Fisher Scientific, Waltham, MA, USA). The surface roughness and wettability of each sample was measured with atomic force microscopy (AFM; Nanoscope V, Veeco, Plainview, NY, USA) and three random 5 μm^2^ areas were calculated as the average surface. Wettability of each sample was examined by an optical contact angle (CA) measuring device (Model OCA15pro, Dataphysics Co., Ltd., Filderstadt, Germany). Each measurement was conducted in triplicate.

### 4.4. BSA Releasing Assay

BNT and BHNT were immersed in 1 mL PBS at 37 °C in a 24-well plate, and the concentrations of solution with released BSA were measured by BCA protein assay kit (Thermo Fisher Scientific, Pierce, Rockford, IL, USA) at specific time points (30 min, and every hour from 1 to 9 h). The decreasing solution volume and the quality of released BSA during each measurement were considered in the analysis.

### 4.5. Cell Culture

The human gingival fibroblasts (HGFs, CRL-2014; ATCC, Manassas, VA, USA) were cultured in Dulbecco’s Modified Eagle’s Medium-High Glucose (DMEM-HG; Thermo Fisher Scientific, Waltham, MA, USA) in the presence of 10 vol% fetal bovine serum (Thermo Fisher Scientific, Waltham, MA, USA) and 1 vol% penicillin/streptomycin (PS, Thermo Fisher Scientific, Waltham, MA, USA) and were used at passage numbers 3–5. The cell suspension was placed in a cell culture plate and incubated at 37 °C and 5% CO_2_. After 24 h, all media were removed, the cultures were washed gently with warm PBS to remove non-adherent cells, and another 10 mL media was added into the culture. All media were changed every other day.

### 4.6. Cell Morphology

To investigate the morphology of HGFs at the early stage of cell adhesion, cells were seeded onto each sample with a density of 1 × 10^4^ cells/well and incubated for 1 and 4 h. Samples were then rinsed with PBS, fixed with 2.5% glutaraldehyde for 2 h at 4 °C, and then washed three times in PBS for 1 h. An ethanol concentration gradient of 30, 50, 75, 90, 95, and 100 *v*/*v*% was used sequentially to dehydrate the samples. All samples were dried for 4 h, sputter-coated with gold, and observed under a thermal field emission environmental SEM.

### 4.7. Early Adhesion of HGFs

HGFs (1 × 10^5^ cells/well) were seeded on each sample for 1 and 4 h, then samples were fixed with 4% paraformaldehyde at room temperature for 15 min, and then were washed three times with PBS. The cell nuclei were stained with DAPI (ZLI-9557, ZSGB-BIO, Beijing, China) for 5 min, then samples were observed and imaged by confocal laser scanning microscopy (Zeiss 710, Jena, Germany). ImageJ software (v1.51 k, Rasband, W.S., ImageJ, US National Institutes of Health, Bethesda, MD, USA) was used to count the amount of adherent HGFs at several time points. The cell density was used to examine the early adherent differences within each sample.

(2) $$Cell\;density=\frac{\left(number\;of\;adherent\;HGFs\right)}{\left(8\;inches\;*\;8\;inches\right)}\;*\;100\%$$

### 4.8. Real-Time PCR

The quantitative real-time polymerase chain reaction was used to investigate the gene expression levels (COL-1, ITGα2, ITGβ1, FAK, and VCL) of HGFs at the early adhesion stage for each sample. HGFs were seeded on samples at a density of 1 × 10^6^ cells/well in a 6-well plate. After incubation at 37 °C and 5% CO_2_, TRIzol reagent (Invitrogen, Carlsbad, CA, USA) was used to extract the total RNA, and then RNA was reverse transcribed into cDNA by using the SYBR GREEN assay. The expression level of each gene was quantified by qRT-PCR analysis with the SYBR Premix Ex Taq II (Takara, Shiga, Japan) on the Bio-Rad CF X Manager system (Bio-Rad, Hercules, CA, USA). Expression levels of the selected genes were normalized to the housekeeping gene GAPDH. Each program was performed in triplicate for each gene.

### 4.9. Statistical Analysis

All experiments were conducted in triplicate. Statistically significant differences (*p* < 0.05) were measured using one-way ANOVA (SPSS18.0, Armonk, NY, USA).

## 5. Conclusions

TNTs were widely used as drug carriers with therapeutic potential. H_2_-TNTs had higher loading efficiency than TNTs. BSA as a common carrier could transfer and load different substances, which could affect cell adhesion and bioactivities of cells. BSA-loaded H_2_-TNTs had nanoscale roughness and a highly hydrophilic surface, however, inhibit the early adhesion of human gingival fibroblasts, which was due to the sudden burst release of BSA at the early stage. After 4 h, with the release of BSA, the inhibiting effect of BSA-loaded H_2_-TNTs gradually decreased over time.

## Figures and Tables

**Table 1:** Proportion of Ti2p (titanium peak), O1s (oxygen peak), C1s (carbon peak), S2p (sulfur peak), N1s (nitrogen peak) of each specimen (%).

	NT	BNT	HNT	BHNT
C1s	24.05	44.72	20.7	44.72
Ti2p	23.89	10.07	25.97	10.07
O1s	52.06	31.46	53.33	30.55
S2p	–	0.86	–	0.67
N1s	–	12.9	–	11.6

**Table 2:** Surface roughness and water contact angles of the samples.

Groups	Roughness (nm)	Contact Angles (°)
NT	98.0 ± 4.8	46.2 ± 3.2
BNT	122.1 ± 5.2	36.4 ± 3.1 ^a^
HNT	98.7 ± 3.4	7.5 ± 0.6 ^ab^
BHNT	148.7 ± 1.5 ^a^	9.4 ± 0.3 ^ab^

Note: data are expressed as mean ± SD (*n* = 3). ^a^ Compared with NT, *p* < 0.05. ^b^ Compared with BNT, *p* < 0.05.

## Data Availability

Data in this article are available on request from the corresponding author.

## Sample Availability

Samples of the compounds are available on request from the corresponding author.

## References

[1] Zhuang L.F., Watt R.M., Mattheos N., Si M.-S., Lai H.-C., Lang N.P. (2016). Periodontal and peri-implant microbiota in patients with healthy and inflamed periodontal and periimplant tissues. Clin. Oral Implants Res..

[2] Takamori Y., Atsuta I., Nakamura H., Sawase T., Koyano K., Hara Y. (2017). Histopathological comparison of the onset of peri-implantitis and periodontitis in rats. Clin. Oral Implants Res..

[3] Wang Y., Zhang Y., Miron R.J. (2015). Health, Maintenance, and Recovery of Soft Tissues around Implants. Clin. Implants Dent. Relat. Res..

[4] Igarashi K., Nakahara K., Kobayashi E., Watanabe F., Haga-Tsujimura M. (2018). Hard and soft tissue responses to implant made of three different materials with microgrooved collar in a dog model. Dent. Mater. J..

[5] Gristina A.G. (1987). Biomaterial-centered infection: Microbial adhesion versus tissue integration. Science.

[6] Zhu Y., Zhang C., Gu Y., Shi J.-Y., Mo J.-J., Qian S.-J., Qiao S.-C., Lai H.-C. (2020). The responses of human gingival fibroblasts to magnesium-doped titanium. J. Biomed. Mater. Res. Part A.

[7] Nevárez-Martínez M.C., Mazierski P., Kobylański M.P., Szczepańska G., Trykowski G., Malankowska A., Kozak M., Espinoza-Montero P.J., Zaleska-Medynska A. (2017). Growth, Structure, and Photocatalytic Properties of Hierarchical V_2_O_5_-TiO_2_ Nanotube Arrays Obtained from the One-step Anodic Oxidation of Ti-V Alloys. Molecules.

[8] Shuang S., Zhang Z. (2018). The Effect of Annealing Treatment and Atom Layer Deposition to Au/Pt Nanoparticles-Decorated TiO_2_ Nanorods as Photocatalysts. Molecules.

[9] Gulati K., Ivanovski S. (2017). Dental implants modified with drug releasing titania nanotubes: Therapeutic potential and develop-mental challenges. Expert Opin. Drug Deliv..

[10] Liu X., Zhou X., Li S., Lai R., Zhou Z., Zhang Y., Zhou L. (2014). Effects of titania nanotubes with or without bovine serum albumin loaded on human gingival fibroblasts. Int. J. Nanomed..

[11] Mei S., Wang H., Wang W., Tong L., Pan H., Ruan C., Ma Q., Liu M., Yang H., Zhang L. (2014). Antibacterial effects and biocompatibility of titanium surfaces with graded silver incorporation in titania nanotubes. Biomaterials.

[12] Ma Q., Mei S., Ji K., Zhang Y., Chu P.K. (2011). Immobilization of Ag nanoparticles/FGF-2 on a modified titanium implant surface and improved human gingival fibroblasts behavior. J. Biomed. Mater. Res. Part A.

[13] Lu R., Wang C., Wang X., Wang Y., Wang N., Chou J., Li T., Zhang Z., Ling Y., Chen S. (2018). Effects of hydrogenated TiO_2_ nanotube arrays on protein adsorption and compatibility with osteoblast-like cells. Int. J. Nanomed..

[14] Wang C., Wang X., Lu R., Gao S., Ling Y., Chen S. (2021). Responses of human gingival fibroblasts to super hydrophilic hydrogenated titanium dioxide nanotubes. Colloids Surf. B Biointerfaces.

[15] Klok O., Munoz A.I., Mischler S. (2020). An Overview of Serum Albumin Interactions with Biomedical Alloys. Materials.

[16] Roufegarinejad L., Jahanban-Esfahlan A., Sajed-Amin S., Panahi-Azar V., Tabibiazar M. (2018). Molecular interactions of thymol with bovine serum albumin: Spectroscopic and molecular docking studies. J. Mol. Recognit..

[17] Valero C.V., Juan A.O., Muñoz A.I. (2010). Adsorption of bovine serum albumin on CoCrMo surface: Effect of temperature and protein concentration. Colloids Surf. B Biointerfaces.

[18] Jahanban-Esfahlan A., Panahi-Azar V. (2016). Interaction of glutathione with bovine serum albumin: Spectroscopy and molecular docking. Food Chem..

[19] Dee K.C., Puleo D.A., Bizios R. (2002). An Introduction to Tissue-Biomaterial Interactions.

[20] Planell J.A., Navarro M., Altankov G., Aparicio C., Engel E., Gil J., Ginebra M.-P., Lacroix D. (2010). Materials Surface Effects on Biological Interactions.

[21] Kim D., Macak J.M., Schimidt-Stein F., Schmuki P. (2008). Capillary effects, wetting behavior and photo-induced tube filling of TiO_2_ nanotube layers. Nanotechnology.

[22] Rabe M., Verdes D., Seeger S. (2011). Understanding protein adsorption phenomena at solid surfaces. Adv. Colloid Interface Sci..

[23] Talha M., Ma Y., Kumar P., Lin Y., Singh A. (2019). Role of protein adsorption in the bio corrosion of metallic implants—A review. Colloids Surf. B Biointerfaces.

[24] Khosa M., Ullah A. (2018). Mechanistic insight into protein supported biosorption complemented by kinetic and thermodynamics perspectives. Adv. Colloid Interface Sci..

[25] Yan Y., Yang H., Su Y., Qiao L. (2015). Albumin adsorption on CoCrMo alloy surfaces. Sci. Rep..

[26] Silva-Bermudez P., Rodil S. (2013). An overview of protein adsorption on metal oxide coatings for biomedical implants. Surf. Coat. Technol..

[27] Kasemo B. (2002). Biological surface science. Surf. Sci..

[28] Pierres A., Benoliel A.-M., Touchard D., Bongrand P. (2008). How Cells Tiptoe on Adhesive Surfaces before Sticking. Biophys. J..

[29] Pierres A., Benoliel A.M., Bongrand P. (2002). Cell fitting to adhesive surfaces: A prerequisite to firm attachment and subsequent events. Eur. Cells Mater..

[30] Fathyunes L., Khalil-Allafi J., Sheykholeslami S.O.R., Moosavifar M. (2018). Biocompatibility assessment of graphene oxide-hydroxyapatite coating applied on TiO_2_ nano-tubes by ultrasound-assisted pulse electrodeposition. Mater. Sci. Eng. C Mater. Biol. Appl..

[31] Beningo K.A., Dembo M., Kaverina I., Small J.V., Wang Y.-L. (2001). Nascent Focal Adhesions Are Responsible for the Generation of Strong Propulsive Forces in Migrating Fibroblasts. J. Cell Biol..

[32] Margel S., Vogler E.A., Firment L., Watt T., Haynie S., Sogah D.Y. (1993). Peptide, protein, and cellular interactions with self-assembled monolayer model surfaces. J. Biomed. Mater. Res..

[33] Rozario T., DeSimone D.W. (2010). The extracellular matrix in development and morphogenesis: A dynamic view. Dev. Biol..

[34] Hynes R.O., Yamada K. (1982). Fibronectins: Multifunctional modular glycoproteins. J. Cell Biol..

[35] Seong J., Tajik A., Sun J., Guan J.-L., Humphries M.J., Craig S.E., Shekaran A., García A.J., Lu S., Lin M. (2013). Distinct biophysical mechanisms of focal adhesion kinase mechanoactivation by different extracellular matrix proteins. Proc. Natl. Acad. Sci. USA.

[36] Shih Y.-R.V., Tseng K.-F., Lai H.-Y., Lin C.-H., Lee O.K. (2011). Matrix stiffness regulation of integrin-mediated mechanotransduction during osteogenic differentiation of human mesenchymal stem cells. J. Bone Miner. Res..

[37] Wang K., Shi L., Linthicum W., Man K., He X., Wen Q., Rojanasakul L.W., Rojanasakul Y., Yang Y. (2019). Substrate Stiffness-Dependent Carbon Nanotube-Induced Lung Fibrogenesis. Nano Lett..

[38] Zamir E., Geiger B. (2001). Molecular complexity and dynamics of cell-matrix adhesions. J. Cell Sci..

[39] Bays J.L., DeMali K.A. (2017). Vinculin in cell-cell and cell-matrix adhesions. Cell Mol. Life Sci..

